# Plants' Metabolites as Potential Antiobesity Agents

**DOI:** 10.1100/2012/436039

**Published:** 2012-05-15

**Authors:** Najla Gooda Sahib, Nazamid Saari, Amin Ismail, Alfi Khatib, Fawzi Mahomoodally, Azizah Abdul Hamid

**Affiliations:** ^1^Faculty of Food Science and Technology, Universiti Putra Malaysia, Selangor, 43400 Serdang, Malaysia; ^2^Faculty of Medicine and Health Sciences, Universiti Putra Malaysia, Selangor, 43400 Serdang, Malaysia; ^3^Laboratory of Natural Products, Institute of Bioscience, Universiti Putra Malaysia, Selangor, 43400 Serdang, Malaysia; ^4^Department of Health Sciences, Faculty of Science, University of Mauritius, 230 Reduit, Mauritius

## Abstract

Obesity and obesity-related complications are on the increase both in the developed and developing world. Since existing pharmaceuticals fail to come up with long-term solutions to address this issue, there is an ever-pressing need to find and develop new drugs and alternatives. Natural products, particularly medicinal plants, are believed to harbor potential antiobesity agents that can act through various mechanisms either by preventing weight gain or promoting weight loss amongst others. The inhibition of key lipid and carbohydrate hydrolyzing and metabolizing enzymes, disruption of adipogenesis, and modulation of its factors or appetite suppression are some of the plethora of targeted approaches to probe the antiobesity potential of medicinal plants. A new technology such as metabolomics, which deals with the study of the whole metabolome, has been identified to be a promising technique to probe the progression of diseases, elucidate their pathologies, and assess the effects of natural health products on certain pathological conditions. This has been applied to drug research, bone health, and to a limited extent to obesity research. This paper thus endeavors to give an overview of those plants, which have been reported to have antiobesity effects and highlight the potential and relevance of metabolomics in obesity research.

## 1. Introduction

Obesity is a rapidly growing epidemic worldwide, presenting an increase in the risk of morbidity and mortality in many countries across the world [1]. Today more than 1.1 billion people are overweight worldwide and 312 million are classified as obese [2]. The World Health Organization (WHO) defines obesity as an abnormal or excessive fat accumulation detrimental to human health. Complications associated with obesity, such as hypertension, hyperlipidemia, diabetes mellitus, cardiovascular disease, cancer, and metabolic disorders are forcing researchers to come up with long-term solutions for weight management and control [3, 4]. Obesity has also been defined as an increased adipose tissue mass, which is the result of an enlargement in fat cells and/or an increase in their number [5]. A crude measure of obesity is the Body Mass Index (BMI), calculated as body weight in kilogram divided by the square of height in meters. Being overweight is defined as a BMI of 25.0–29.9 kg m^−2^, and a BMI exceeding 30 kg m^−2^ is considered as obese. An extreme obesity is defined as a BMI of greater than 40 kg m^−2^ [6]. Today, more than 65% of adults in the United States are overweight or obese [7]. In developing countries like Malaysia, 23% of the adult population was found to be overweight and 14% obese [8].

The main cause of obesity is exceeding energy input over energy expenditure. Our genetic build-up, to a certain extent, plays a role in determining whether we will suffer from obesity or not at some point of our life. While it is easy to isolate the gene responsible for obesity in rodents, it is not the case for humans. About only 33% of the variance in body weight is due to genetic influences but environmental influences are of great importance. These factors include lifestyles and socioeconomic factors [9]. The adipose tissue is not a single entity but consists of several subclasses such as the visceral and subcutaneous layers, which have different implications for health. The adipose tissue is not only a storage organ for triacylglycerides but also an endocrine organ where numerous chemical messengers called adipokines are released for better communication with other tissues [10].

Although reduction of caloric intake by diet and increased level of physical activity are very well-known approaches to lose weight, the needs for drugs and other supplements are fast gaining acceptance. Many diets have been advocated for weight loss but there is little scientific evidence to recommend one diet over another [6]. Due to the inconsistent effort in achieving a negative energy balance through diet and exercise, the needs for drugs and other supplements are fast gaining acceptance. However, drug discovery for antiobesity agents have long been plagued with inconsistency and side effects. The current clinical treatment for obesity is a synthetic analogue of Lipstatin, Orlistat. Orlistat is reported to be a potent inhibitor of gastric, pancreatic, and carboxylester lipase [11]. Orlistat is also a gastrointestinal lipase inhibitor that competes with dietary fats for sites on the lipase molecules and has been shown to block the absorption of about 30% of dietary fat at a therapeutic oral dose of 120 mg three times a day. Orlistat does not show any apparent effect on appetite [12] but is known to inhibit several human digestive and metabolic lipases. Orlistat has been defined as an active site-directed inhibitor that reacts with the nucleophilic serine residue from the catalytic triad of pancreatic lipase (PL) [11]. By covalently blocking the active site, Orlistat inhibits the hydrolysis of dietary triglycerides and thus reduces the subsequent intestinal absorption of the lipolysis products monoglycerides and free fatty acids. Orlistat also inhibits gastric lipase [13] and lipoprotein lipase (LPL) [14] in vitro. Other means of preventing obesity is through appetite suppression, modulation of adipocytes proliferation and differentiation, the adipogenic factors, increase in thermogenesis, or inhibition of fatty acid synthase (FAS).

The role of medication in weight loss is controversial and lits effectiveness appears too limited. The Food and Drug Administration (FDA) has approved no weight loss drug for use for more than 2 years. Hence drugs represent a short-term solution for a long-term problem with only modest benefits and unclear risk(s) [6]. Diets high in fats tend to promote obesity, hence inhibition of digestion and absorption of dietary fats is a logical remedy in treating obesity. As synthetic drugs fail to give desired effects and with side effects involved, the utilization of traditional and alternative medicines is fast gaining acceptance. According to the WHO, in developing countries, medicinal plants contribute significantly to primary health care and herbal medicine trade is worth US$ 60 million. With more and more people rejecting chemical drugs due to the fear of side effects, natural-based products are fast gaining popularity, thus justifying extensive research in this field. The last decade has seen a lot of improvements in advanced methods used in systems biology research. The “omics” technology: genomics, proteomics, transcriptomics, and lately metabolomics have made it possible to validate the use to traditional medicines scientifically, identify new bioactive compounds, elucidate their mechanisms of action, and assess toxicity and quality control [15]. This paper aims to discuss plants that have been reported for antiobesity effects and potential of metabolomics in antiobesity research. Plants can be grouped based on different mechanisms of action: enzymes inhibition, modulation of adipogenesis & adipogenic factors, and appetite suppression.

## 2. Enzymes Inhibition

Inhibition of the digestion and absorption of dietary fat has been used as target in obesity treatment [16]. It is understood that PL is the most important enzyme responsible for the digestion of triglycerides into mono and diglycerides and in smaller fatty acids to be absorbed by the body. Researchers and health professionals support that an inhibition of PL can reduce digestion of fats, hence their assimilation and absorption. This can mimic a reduced calorie intake in obese patients and help in preventing additional weight gain [4]. Lipoprotein lipase (LPL) has also been targeted in obesity as it has been found out that there is an increase in LPL level in obese subjects. Since LPL catalyzes the hydrolysis of blood triglycerides to release free fatty acids (FFA) and hence increases the storage of triglycerides in adipose tissue [17], inhibition of LPL is expected to reduce assimilation of FFA and help in controlling obesity. Inhibition of enzymes involved in carbohydrates digestion and metabolism are preferentially studied in the treatment of type 2 diabetes mellitus. However, it can also be considered and relevant in obesity research, owing to the fact that carbohydrates are major sources of calories in the human diets. Those enzymes inhibitors slow down carbohydrate metabolism and subsequently reducing postprandial hyperglycemia, hence delaying the conversion of glucose in the adipose tissue to triacylglycerol [18].

A panoply of botanicals have been assessed for their antilipase activity as a therapy to manage obesity. A comprehensive review on lipase inhibitors from natural sources was published recently [19]. Natural PL inhibitors such as saponins, polyphenols, terpenes, and microbial by-products have been described as unexplored potential in the management of obesity and new drug discovery. The antiobesity effect of CT-II, an extract from the edible Nomame herb, was reported. This extract was found to inhibit porcine PL in vitro in a dose-dependent manner. A very low concentration of 0.1 mg/mL of the extracts resulted in 50% inhibition of lipase activity. The feeding of CT-II extract inhibited weight gain and plasma triglyceride levels in lean rats fed a high-fat diet, without affecting food intake. In obese subjects, after 6 months of feeding, weight and total body fat were significantly lost and additional weight gain was suppressed. Hence, it was concluded from this study that CT-II might be a powerful lipase inhibitor and could be used as a weight control in obese subjects [20].

Teas have been long used in Chinese traditional medicine to treat various ailments such as obesity and lipids disorders. It is now known that tea saponins, predominantly present in Oolong tea, have a good inhibitory effect on PL activity, validating the popularity of teas in slimming diets. Tea saponin, a pancreatic lipase inhibitor, was also found to suppress increase in body weight, adipose tissue weight, and diameter of adipocytes of rodents fed a high-fat diet. The excretion of triacylglycerol content in the feces was also increased. The authors suggested that the antiobesity effects of tea saponins may be mediated through absorption of dietary fat through an inhibition of PL activity [21]. A standardized *Camellia sinensis* L. based drug, AR25 (Exolise), was proposed as an antiobesity agent. The green tea extract, standardized at 25% catechin, had direct inhibition on gastric and pancreatic lipases as well as stimulation of thermogenesis. The effect of green teas extract was also evaluated in moderately obese patients. In the clinical trial, after 3 months of consumption of 375 mg catechins, body weight was decreased by 4.6% and waist circumference by 4.48%. The results showed that Exolise, with the active component being catechin, can be a natural antiobesity agent, through its antilipase activity and increase in thermogenesis [22].

*Salacia reticulata* is a plant of Indian origin that is commonly used in Japan as antiobesity and anti-diabetic agent. A hot-water-soluble extract of *S. reticulata *(SRHW) was investigated for its antiobesity effect, both in vitro and in vivo. SRHW inhibited pancreatic lipase, LPL, and glycerophosphate dehydrogenase in vitro. The extract showed no effect on hormone sensitive lipase. Female Zuker obese rats, fed on SRHW showed a significant decrease in body weight and fat storage, whereas no significant difference was noted in males. The antiobesity effect of SRHW was attributed to high concentration of polyphenols such as mangiferin, catechins, and condensed tannins [23].

Grape seed extract (GSE) has also been found to inhibit LPL in vitro [24]. GSE inhibited PL activity dose dependently, with maximum inhibition of 80%. At a concentration of 1 mg/mL, GSE inhibited LPL activity by 30%. Peanut shell extracts (PSEs) were found to inhibit various lipases such as PL, LPL, and hormone-sensitive lipase (HSL). Rats fed on PSE showed increased fecal excretion of fats, and weight gain was prevented in rats fed on high diet supplemented with 1% PSE. The increased fecal excretion of lipids suggests that PSEs work by inhibiting various lipases and might be useful in inhibiting fat absorption [25].

There is emerging evidence that soy proteins can reduce body weight. As reviewed by Bhatena and Velaquez [26], the antiobesity effect of soy protein and its isoflavones appears to be due to the modulation of pancreatic insulin and antioxidative actions and through long-term substitution of animal proteins by vegetable proteins in low-calorie diet. A tetrapeptide in soy protein isolate and hydrolysate showed antiobesity effect in genetically obese mice by reducing perirenal fat mass and plasma glucose level. The beneficial effects of this protein food are attributed to its high content of isoflavones mainly genistein and daidzein [26].

We recently reported the inhibitory effect of selected tropical plants from Malaysia on lipoprotein lipase activity in vitro. Extracts of *Morinda citrifolia* fruit (noni), *Momordica charantia* (bitter gourd), and *Centella asiatica* (Asian pennywort) were found to inhibit the activity of LPL in vitro. At a concentration of 0.1 mg/mL, Noni fruit extract showed the highest inhibition of 21.5%, followed by Asian pennywort extract (18.2%) and bitter gourd extracts (10%). Though the complete mechanism of inhibition was not reported, it was suggested that the synergistic effect of several flavonoids such as catechins and epicatechin might have been responsible for this bioactivity [27].

## 3. Inhibitors of Adipogenesis and Adipogenic Factors

White adipose tissue (WAT) is the main energy reserve in humans and animals, where excess energy is stored as triglycerides. The balance between energy intake and energy output, defined as energy homeostasis and control of body weight, depends on the equilibrium of this process. An imbalance between energy intake and energy expenditure has led to rapid weight gain. The central nervous system, most specifically the hypothalamus, regulates and integrates food intake and energy expenditure, whereas peripheral tissues such as the muscles and adipose tissue deals with nutrient metabolism and energy production [28]. A complex network consisting of long-term and short-term signals have been reported to regulate energy intake. These signals are integrated by the hypothalamus and involve orexigenic and anorexigenic neuropeptides that control appetite and metabolism [29].

During a period of energy deprivation or starvation, triacylglycerols are mobilized to compensate for the lack of energy. An excess of WAT has been thought to be one of the major culprit in the prevalence of obesity in today's world [30]. It is known that the growth of the adipose tissue involves the formation of new adipocytes from precursor's cells leading to an increase in the size of the adipocyte. Treatment that regulates the size and the number of the adipocytes and the expression of signals involved in energy balance and the inhibition or enhancement of specific adipokines have been suggested to express antiobesity-related bioactivities [31].

The most common plant implicated in weight loss is without any doubt green tea. There are various studies promoting the use of green teas as slimming aids. Recently, the effect of green tea epigallocatechin gallate (EGCG) was shown to inhibit the proliferation and differentiation in the primary human visceral preadipocytes. The study reported that the ability of EGCG to promote weight loss can be partly due to its ability to suppress the number of adipocytes and also suppress triacylglycerols uptake [32]. In another study, supplementation with tea catechins resulted in significant reduction in body weight induced by a high-fat diet as well as liver and visceral fat accumulation. The stimulation of the hepatic lipid metabolism by the catechin present in the tea was suggested to be responsible for the antiobesity effect. Hence the long-term consumption of tea might be beneficial for the suppression of diet-induced obesity [33]. Extracts of *Momordica charantia*, *Centella asiatica,* and *Morinda citrifolia* inhibited the proliferation on 3T3-L1 preadipocytes in a dose-dependent manner with *Momordica charantia* having the most toxic effect with an LC_50_ value of 1.7 mg/mL, followed by *Centella asiatica* with an LC_50_ of 2.72 mg/mL. These two extracts also inhibited the differentiation of preadipocytes in a time-and dose-dependent manner. These antiobesity-related bioactivities were again attributed to the potential synergistic effects of flavonoids [27].

Perilla oil, which is rich in (*n*-3) polyunsaturated fatty acids, was found to prevent the excessive growth of visceral adipose tissue in rats by a downregulation of adipocyte differentiation. Perilla oil was found to suppress the late phase of adipocyte differentiation and prevention increase in lipoprotein expression in rats. It was suggested that the utilization of Perilla oil, rich in *α*-Linolenic acid chemically stable, and hence its consumption is good for human to prevent the development of visceral adipose tissue in obese patients [34].

*Hibiscus sabdariffa* L., a medicinal plant widely used as beverage, had also been reported to modulate negatively on obesity. Studies carried out proved that water extract of this plant inhibits the adipocyte differentiation through a modulation of the P13-K and MAPK pathways that are critical for adipogenesis to happen [35]. Hibiscus extracts were also shown to inhibit porcine pancreatic amylase, effective in decreasing levels of cholesterol, lipids, and triglycerides in rats and inhibitory effect on adipogenesis in 3T3-L1 preadipocytes [36]. The traditional knowledge combined with recent scientific findings suggests that extracts of hibiscus can be used as a functional food or alternative medicinal beverages in the management of obesity.

Lemon has also been shown to suppress diet-induced obesity by suppressing body weight gain and body fat accumulation. The lemon polyphenols have been shown to be antiobesity in action by increasing beta oxidation through upregulation of mRNA level in liver and WAT [37]. An aqueous extract of *Salacia reticulata* and cyclodextrin was found to reduce the accumulation of visceral fat mass, plasma triglyceride, and body weight in animals with high fat diet induced obesity. The anti obesity effects were attributed to inhibition of carbohydrate and lipid absorption from small intestine [38]. The effect of lanostane triterpenes from fruiting bodies of *Ganoderma lucidum *was investigated on adipocyte differentiation in 3T3-L1 cells. As a compound isolated from *Ganoderma lucidum, t*-Butyl lucidenate B has been found to reduce accumulation of triglycerides in 3T3-L1 adipocytes by 72%. The compound also suppressed GPDH activity in cells, as well as gene expression of PPAR-*γ*, C/EBP-*α* and SREBP-1c dose dependently [39]. The antiobesity-related bioactivity of Asian spices was recently reported [40]. Thirty spices commonly consumed in Asia were assessed for their antiobesity potential through different in vitro assays: adenosine A1 receptor binding, cannabinoid CB1 binding, and TNF-*α* and 3T3-L1 adipocytes differentiation induction. Results revealed that sesame seed and red chili had high binding activity to the adenosine A1 receptor, nutmeg, mace, black pepper and turmeric binds predominantly to the cannabinoid CB1 receptor, while piment and turmeric showed high inhibition of TNF-*α* accumulation. Major pure compounds isolated from the selected spices failed to give similar results, prompting the researchers to conclude that antiobesity bioactivities of those spices might be attributed to the minor compounds or a synergistic effect between compounds [40].

## 4. Appetite Suppressants

Fatty acid synthase (FAS) is known to catalyze the reductive synthesis of long chain fatty acids from acetyl coenzyme A and malonyl-CoA. It is proven that an inhibition of FAS can reduce food intake and body weight in mice treated with FAS inhibitors. This makes the inhibition of FAS a potential therapeutic target to suppress appetite and induce weight loss [41]. Many plants and their products have been shown to inhibit FAS and hence impact negatively on appetite. Epigallocatechin gallate from green tea was found to be a strong inhibitor of FAS from chicken liver both by reversible fast binding and irreversible slow binding. The inhibitory effects were comparable to that of established FAS inhibitors such as cerulenin and C75, a synthetic FAS inhibitor [42]. A novel compound from South Africa succulent of the Hoodia family plant has been documented to reduce appetite in animals without any sedentary effect. The compound indentified as P57 was also tested clinically where it showed positive results in suppressing appetite and reduced body weight in obese individuals [43]. In particular, extracts from *Hoodia pilifera* and *Hoodia gordonii* were characterised as possessing appetite suppressing properties. Oral gavage of different compounds and doses (6.25–50 mg/kg) resulted in decreased food consumption. Compared to fenfluramine control sample, reduction in food intake by compounds in Hoodia species was greater [44]. *Caralluma fimbriata,* an edible cactus used by tribal Indians to suppress hunger and increase endurance, has shown to be antiobesity in action. Fifty men and women ingested 1 g of Caralluma extract per day for 60 days. Compared to placebo, the extract appears to suppress appetite and reduce waist circumference significantly. Feeding of the extract also resulted in a decrease in body weight, body mass index, hip circumference, and body fat in overweight patients [45]. *Catha edulis,* commonly known as khat, is usually chewed after meals in some African communities. It is known to decrease feeling of hunger and increase feeling of satiety due to release of cathinone, which suppresses appetite. Hormones ghrelin and peptide YY (PYY) are released with hunger and after meal, respectively, and are known to modulate appetite. The effect of usual khat chewing for three hours two times was investigated in 6 individuals. The plasma samples were then analysed for cathinone, ghrelin, and PYY. Results showed that there was no effect in ghrelin and PYY levels, suggesting that other mechanisms might be involved in the observed suppression of appetite. On the other hand, there was an increase in cathinone levels. Cathinone is explained to have a positive correlation with satiety and a negative correlation with the feeling of hunger [46]. *Garcinia cambogia* is already available on the market as a dietary supplement to lose weight. The primary component of the fruit rind is (-)-hydroxycitric acid (HCA), and supplementation in experimental animals has showed that HCA can suppress appetite and inhibit body fat biosynthesis. In rat brain cortex, HCA was shown to increase availability of 5-hydroxytryptamine or serotonin. This neurotransmitter is implicated in the appetite regulation and control [47]. A syngergestic effect of different herbs to promote weight loss has also been suggested. A mixture of herbs (green tea, *Coleus forskohlii*, yerba mate and *Betula alba*) gave a more potent antiobesity effect as compared to the single entity. The mixture of plants was better than separate plants in lowering body weight and suppress food intake in obese rats. The mechanism of action was reported to be an increase in thermogenesis, antioxidative effect, and the inhibition of adipocyte proliferation amongst others [48].

## 5. Dietary Phytochemical Strategies for Obesity and Obesity-Linked Diseases Using Metabolomic Approaches

Metabolomic is an emerging field that promises the ability to personalize diet in improving health, preventing disease states, and improving performances. It is defined as the quantitative measurement of all metabolites in an organism at a specified time, under specific environmental conditions. Metabolites reflect the cellular processes that control the biochemical reactions of the cell, tissue, or whole organism [49]. Measurements of these metabolites will reveal the biochemical status of an organism and in turn can be used to monitor and assess gene function. It is established that improved technology such as proteomics, transcriptomics, and genomics offer the platform to bridge traditional medicine and molecular pharmacology [50], assess bioactivity of traditional products [15], and study the biochemical effects on bioactive compounds on human subjects [51]. Metabolomics is concerned with the high throughput identification and quantification of small molecule (<1500 Da) metabolites [52] and has hence emerged as a more robust approach in predicting gene activity as affected by interaction with foods, than the currently more widely used transcriptomic and proteomic approaches. Gene expression and proteomic data only indicate potential of physiological changes. Data generated delivered interesting insights for the understanding of metabolic responses of human as affected by dietary interventions [53].

Most of the work in metabolomics has focused on clinical or pharmaceutical applications such as drug discovery, drug assessment, clinical toxicology, and diseases states [54]. However, over the past few years, metabolomics has also emerged as a field of increasing interest to food and nutrition scientists [55]. With regard to natural product research, metabolomics in the context of systems biology is seen as a bridge linking Traditional Chinese Medicine (TCM) and molecular pharmacology [50]. Metabolomics has been applied to improve identification of bioactive metabolites from extracts [56], to study the biochemical effects of bioactive compounds in human subjects [50], for quality control of phytomedicines [56], and to assess their toxicity [57]. One of the first studies to use metabolomic techniques in a diet-intervention trial involved studing the effects of soy-derived proteins and soy isoflavones in healthy premenopausal women under controlled environmental conditions [51]. NMR and chemometric techniques were used to analyze the blood plasma profile changes in premenopausal women on a multiweek soy diet. The results showed that significant differences could be detected in lipoproteins, amino acid, and carbohydrate profiles that related to metabolic pathways responsible for osmolyte fluctuation and energy metabolism. A similar study by Fardet and colleagues [58] explored the influence of whole-grain and wheat flour diets on rats using NMR-based methods. These studies showed that the whole-grain-fed animals had higher urinary levels of Kreb's cycle intermediates, aromatic acids, and hippuric acid. NMR studies on plasma and liver showed increases in glutathione (reduced) and betaine, which are general indicators of good redox status and lower oxidative stress. Others include studies on the effects of fruits and vegetables diversity on the levels of oxidative biomarkers [59], the influence of concentrated red grape juice on oxidative stress, lipidemic, and inflammatory markers in blood [60], the effect of extra virgin olive oil on plasma inflammatory and oxidative stress markers [61], the consequences of macadamia nut consumption on cardiovascular disease [62], and the influence of gazpacho soup consumption on plasma biomarkers of oxidative and inflammatory stress [63]. *Galphimia glauca*, a Mexican plant has been traditionally used to treat diseases of the central nervous system but therapeutic effects of the plants tend to vary with geographical locations. Animals testing reported anxiolytic and sedative activities, with only 2 out of the 6 collections used showing high bioactivity. The metabolic profiling of the extracts was then performed using ^1^H NMR and partial least square-discriminant analysis. Results attributed the bioactivity to the presence of galphimine, present in the 2 active extracts and lacking in the less active remaining 4. There was a strong correlation between anxiolytic and sedative effects and metabolic profiles of plants, showing that metabolomics can be successfully used to identify novel drug compound from pants [64]. In another study, Jiang and coworkers studied the metabolic profiling and phylogenetic analysis of medicinal Zingiber species. The plants species derived from different origin showed no difference in qualitative metabolites although quantitative differences were detected through GC-MS. The anti-inflammatory activities of the different species were also determined. There was a lack of correlation between gingerol found in *Z. officinale* and anti-inflammatory activities, suggesting that other compounds in Zingiber species might be responsible for the anti-inflammatory activities. These compounds remain to be elucidated [65]. The use of metabolomics to prove efficacy and unravel mechanism of action of natural compounds is fast gaining popularity, both in animal and human studies. The effect of a traditional and popular antiageing agent, *Herba epimedii,* was evaluated using ^1^H NMR and PCA techniques. Ten metabolites of interest were identified as antiageing biomarkers in the urine of rats. Further studies carried out after intervention with the flavones of *Herba epimedii* revealed that the urine profile of 24-month-old rats shifted to that of 18-month-old models [66]. In relation to obesity, metabolomics has been used to assess the effect of obesity in a rat model of obesity induced by a high-fat diet. The metabolic changes in the urine of high-fat-diet-induced obese rats using ^1^H-nuclear magnetic resonance spectroscopy-based metabolic assessment were reported. The spectra were analyzed using multivariate statistical analysis to identify the separation of groups. Multivariate data analysis deals with the statistical analysis of thousands of variables at a time. In most cases of metabolomics studies, the number of observations is considerably smaller than the measured variables. The metabolic profile of urine was different in low gainers and high gainers on the same diet. Metabolites of interest include betaine, taurine, acetone/acetoacetate, phenylacetylglycine, pyruvate, lactate, and citrate. This study successfully confirms the accuracy of NMR-based metabolomics to study the effect of diet on obesity [67].

Another study attempted to study childhood obesity from a metabolomic perspective. Zheng and colleagues have recently carried out a plasma metabolic fingerprinting of childhood obesity by GC/MS in conjunction with multivariate statistical analysis. Plasma from normal, overweight and obese children was subjected for metabolic profiling using GC/MS. Different metabolic patterns were revealed as computed by uncorrelated linear discriminant analysis. Isoleucine, glyceric acid, serine, 2,3,4-trihydroxybutyric acid, and phenylalanine were discovered as potential biomarkers for childhood obesity. The authors also reported that waist hip ratio, total triglycerides, total cholesterol, high-density lipoprotein, and low-density lipoprotein were closely related to metabolic perturbations of childhood obesity and advised that these components to be given more importance in the clinical setting rather than BMI alone [68].

In spite of the increasing use of metabolomics techniques in natural health product research, there is limited information available on dietary phytochemical strategies for obesity and obesity-linked diseases using the metabolomics approach. To the best of our knowledge, there is no study reporting the metabolite modulation of obese subjects fed a high-fat diet, supplemented with natural bioactives. But, metabolomics being still in its infancy when it concerns natural product research, there is no doubt that more and more information will become available on antiobesity effects of plants' bioactives, from a metabolic perspective.

## 6. Conclusion

The popularity of alternative medicine is increasing by leaps and bounds as shown by the increase in demand of natural health products. As drugs failed to give desirable long-term results, more and more interest is generated in the use of medicinal plants to alleviate obesity. Various plants and natural products have been assessed for their potential antiobesity effect both in vitro and in vivo. There is increasing proof that plants and their products can exert antiobesity effects through various mechanisms such as antilipase, anti adipogenesis effect, or suppressing appetite. Some researchers also suggest that a synergy between different plants give a better effect in terms of antiobesity as compared to the single plants. The major drawback is the questionable effect in human subjects. Results from in vitro or in vivo animal models cannot be directly extrapolated to human subjects and in many cases are found to be not significantly effective. There is always the risk of toxicity, lack of information on mechanisms of action, and poor quality control of natural herbal antiobesity products. Recent advances in systems biology research, such as metabolomics, have enabled researchers to identify bioactive compounds, assess bioactivity, and elucidate mechanisms of therapeutic action and toxic effects and to maintain quality control more accurately. Medicinal plants as antiobesity agents are gaining more credibility in the scientific community and some of the natural compounds have reached clinical trials (e.g., P57 from* H. gordonii*). Without any doubt, there is a potential of using plants and their products as a therapeutic treatment to manage obesity. There are also thousands of unstudied plants across the world, some of which have traditionally been used to maintain ideal body weight or as slimming agents, thus justifying the need for deeper research in this field. Nonetheless, sound methodologies are the key to provide scientifically valid and sound information so that these plants or compounds make it as antiobesity agents in the clinical setting.
